# Periprothetische Acetabulumfraktur

**DOI:** 10.1007/s00113-025-01639-x

**Published:** 2025-10-02

**Authors:** Jose A. Roshardt, Silviya Ivanova, Hannes Kuttner, Christiane Leibold, Simon D. Steppacher, Moritz Tannast, Johannes D. Bastian

**Affiliations:** Universitätsklinik für Orthopädische Chirurgie und Traumatologie, Inselspital, Universitätsspital Bern, Freiburgstrasse 3010, Bern, Schweiz

**Keywords:** Hüfttotalendoprothese, Implantatstabilität, Revisionsprothetik, Osteosynthese, Algorithmen, Total hip arthroplasty, Implant stability, Revision surgery, Osteosynthesis, Algorithms

## Abstract

Periprothetische Acetabulumfrakturen (PPAF) sind eine seltenere, aber zunehmend relevante Komplikation nach Hüfttotalendoprothesen. Ihre Versorgung ist komplex und erfordert Expertise in der Frakturversorgung und der Revisionsendoprothetik. Die Behandlungsplanung basiert auf den morphologischen Frakturmerkmalen nach dem Pfeilerkonzept nach Letournel, dem Ausmaß von Knochendefekten (frakturbedingt oder vorbestehend), der Stabilität der Pfannenkomponente sowie patientenbezogenen Faktoren. Diese Übersichtarbeit beschreibt einen praxisbezogenen Algorithmus zur Behandlung von PPAF. Neben der präoperativen Diagnostik werden etablierte Klassifikationssysteme, operative Zugangswege und therapeutische Strategien bei unterschiedlichen Fraktursituationen dargestellt.

## Abstract (en)

Periprosthetic acetabular fractures (PPAF) are a rare but increasingly relevant complication following total hip arthroplasty. The management is complex and requires expertise in fracture treatment and revision arthroplasty. The treatment planning is based on the morphological fracture characteristics according to the column concept (Letournel), the extent of bone defects (fracture-related or pre-existing), the stability of the acetabular component and patient-related factors. This review article presents a practice-related algorithm on how to approach PPAFs. In addition to the preoperative diagnostics, established classification systems, surgical approaches and treatment strategies for various fracture scenarios are also presented.

Die periprothetische Acetabulumfraktur (PPAF) ist aktuell eine seltene Komplikation nach einer Hüfttotalendoprothesenimplantation. Die berichtete Inzidenz beträgt 0,07 % und ist deutlich niedriger als die Inzidenz periprothetischer Femurfrakturen, mit einem Verhältnis von ca. 1:50 [1, 2]. Die Behandlung ist anspruchsvoll und erfordert sowohl Expertise in der Frakturversorgung als auch in der Revisionsendoprothetik [3]. Trotz wachsender klinischer Bedeutung ist die Evidenzlage begrenzt und beruht vorwiegend auf Fallserien und retrospektiven Studien [1]. In den letzten Jahren wurden verschiedene Behandlungskonzepte zur Versorgung von PPAF vorgeschlagen. Diese orientieren sich primär an der Ätiologie, sodass das therapeutische Vorgehen bei intraoperativen, traumatischen und pathologischen Frakturen differenziert wird [4]. Zusätzlich werden das Alter und die Stabilität des Implantates, die Knochenqualität sowie patientenspezifische Faktoren berücksichtigt [4–7]. Mit der prognostizierten Zunahme primärer Hüfttotalendoprothesen um bis zu 27 % in Deutschland bis 2040 sowie vergleichbaren Tendenzen in den USA, England und Australien ist das Verständnis der aktuellen Evidenzlage und der bestehenden Behandlungskonzepte essenziell [6, 8]. Ziel dieses Artikels ist es, den aktuellen Kenntnisstand und praxisrelevante Aspekte zu PPAF zusammenzufassen. Folgende Fragen sollen beantwortet werden:

1. Welche präoperative Diagnostik ist relevant?
2. Wie werden PPAF klassifiziert?
3. Wie wird der chirurgische Zugangsweg gewählt?
4. Was ist die Versorgungsstrategie in Abhängigkeit von Frakturdislokation und Implantatstabilität?

## Diagnostik

Periprothetische Acetabulumfrakturen erfordern eine gründliche Anamnese mit Fokus auf die Lebensumstände und Ansprüche der betroffenen Patienten, die Funktionalität der Prothese vor der Fraktur, vorbestehende, Operationen im Situs einschliesslich nicht-orthopädischer Eingriffe und den Unfallmechanismus [9]. Beim Unfallmechanismus kann es sich um ein Hochenergietrauma oder, insbesondere bei älteren Patienten, um ein Niedrigenergietrauma handeln. Letzteres kann bei vorbestehender Implantatlockerung eine PPAF auslösen sowie umgekehrt eine Implantatlockerung ein Sturzereignis verursachen. Bestehende Hüftschmerzen vor dem Trauma können ein Hinweis auf eine aseptische oder septische Lockerung sein [7].

Die CT erlaubt die genaue Analyse der Fraktur und der Implantatstabilität gemeinsam mit Voraufnahmen von konventionellen Röntgen

Vor einer Revisionsoperation sind Entzündungswerte im Blut zu bestimmen [9]. Zur bildgebenden Standarddiagnostik der PPAF gehören eine Beckenübersichtsaufnahme in anterior-posteriorer (a-p.) Projektion und eine axiale Aufnahme der betroffenen Hüfte. Abhängig von den morphologischen Frakturmerkmalen sollten Ala- und Obturatoraufnahmen, evtl. Inlet‑, Outlet-Röntgen ebenfalls ergänzt werden [7]. Zur weiteren Beurteilung ist eine Computertomographie (CT) mit Metallartefaktsuppression erforderlich, idealerweise einschließlich Femur zur Beurteilung der femoralen Schafttorsion. Dies erlaubt eine genauere Frakturanalyse und -klassifikation, v. a. die Einschätzung der Pfeilerintegrität, bestehender Knochendefekte oder -lysen sowie der Stabilität des Implantats [6]. Bei intrapelviner Implantatmigration oder klinischem Verdacht auf vaskuläre Beteiligung ist eine CT-Angiographie zur Darstellung möglicher Gefäßverletzungen indiziert [6].

## Klassifikation

Periprothetische Acetabulumfrakturen werden in intra- und postoperative Frakturen unterteilt. Intraoperative Frakturen entstehen typischerweise während der Implantation oder Entfernung der Pfannenkomponente, insbesondere bei zementfreien Implantaten. Zu den Risikofaktoren zählen u. a. schlechte Knochenqualität, Knochendefekte, Osteolysen und eine Pfannenmalposition (z. B. bei exzessivem Reaming, [4, 10]). Postoperative Frakturen sind meist traumatischer Genese, können aber auch im Kontext von Infektionen, Osteolysen oder Tumoren auftreten. In 0,9 % der Fälle zeigt sich eine pelvine Diskontinuität [6]. Dies entspricht einer vollständigen Unterbrechung der Lastübertragung im Gelenk, von proximal (Os ilium) nach distal (Os ischii, Os pubis).

Zur morphologischen Beschreibung des Frakturverlaufs ist die Klassifikation nach Judet und Letournel empfehlenswert, obwohl sie aus der Acetabulumchirurgie des nativen Gelenks stammt [6]. Sie gliedert das Acetabulum anatomisch im Wesentlichen in einen vorderen und einen hinteren Pfeiler. Frakturen werden in 5 einfache und 5 assoziierte Frakturformen unterteilt. Zu Letzteren zählen Frakturen des hinteren Pfeilers ohne/mit Beteiligung der hinteren Wand, Querfrakturen mit Hinterwandbeteiligung, T‑förmige Frakturen, Frakturen des vorderen Pfeilers mit posterior hemitransversalem Verlauf sowie Frakturen beider Pfeiler [11].

Die Beurteilung des Knochendefekts ist ein wesentlicher Bestandteil der präoperativen Planung bei PPAF. Die Klassifikation der American Academy of Orthopaedic Surgeons (AAOS) nach D’Antonio et al. unterscheidet zwischen kavitären, segmentalen und kombinierten Defekten sowie pelviner Diskontinuität [12]. Kavitäre Defekte betreffen spongiösen Knochen bei erhaltenem Pfannenrand und beiden Pfeilern. Segmentale Defekte weisen einen Substanzverlust an Pfannenrand oder Pfeilern auf. Kombinierte umfassen beides [13].

Zusätzlich kann die Klassifikation nach Paprosky angewendet werden; diese wurde 1994 zur Beurteilung chronischer acetabulärer Defekte bei Revisionsoperationen eingeführt. Sie basiert auf der Richtung und dem Ausmaß der Pfannenmigration, der verbliebenen Knochensubstanz sowie der Möglichkeit zur stabilen Implantatverankerung [14]. Paprosky und Della Valle haben diese Klassifikation im Jahr 2003 erweitert, um komplexe Defekte im Zusammenhang mit periprothetischen Frakturen differenzierter abzubilden (Tab. 1). Die modifizierte Version berücksichtigt neben dem strukturellen Defekt auch den Zeitpunkt der Fraktur (intra- oder postoperativ) [15].

**Tab. 1:** Klassifikation periprothetischer Acetabulumfrakturen gemäß Paprosky und Della Valle [15].

Typ I: intraoperativ (Implantateinbringung)	a: erkannt, Fraktur undisloziert, Implantat stabil
	b: erkannt, Fraktur disloziert, Implantat instabil
	c: intraoperativ nicht erkannt
Typ II: intraoperativ (Implantatentfernung)	a: Knochenstockverlust < 50 %
	b: Knochenstockverlust > 50 %
Typ III: traumatisch	a: stabiles Implantat
	b: instabiles Implantat
Typ IV: spontan	a: Knochenstockverlust < 50 %
	b: Knochenstockverlust > 50 %
Typ V: pelvine Diskontinuität	a: Knochenstockverlust < 50 %
	b: Knochenstockverlust > 50 %
	c: assoziiert mit Beckenbestrahlung

Das Unified Classification System (UCS) wurde 2014 von der Arbeitsgemeinschaft für Osteosynthesefragen (AO) als Erweiterung bestehender Klassifikationen entwickelt, um periprothetische Frakturen verschiedener Lokalisationen in einem einheitlichen Klassifikationssystem zu erfassen. Es berücksichtigt sowohl die morphologischen Frakturmerkmale als auch die Stabilität des Implantats. Die Gelenke sind nummeriert von I (Schulter) bis VI (Sprunggelenk), das Becken entspricht der AO-Region 6. Die PPAF werden als IV/6 kodiert. In Analogie zur Vancouver-Klassifikation beschreibt das UCS die folgenden Frakturtypen: Typ A als extraartikuläre, apophysäre Abrissfrakturen, Typ B als Frakturen im Bereich des Pfannenimplantats (B1 bei festem, B2 bei gelockertem Implantat mit ausreichender Knochensubstanz, B3 bei Knochendefekt), Typ C als implantatferne Frakturen, Typ D als Frakturen im Beckenbereich bei bilateraler Hüftprothese, Typ E als kombinierte periprothetische Acetabulum- und Femurfrakturen, Typ F als Acetabulumfrakturen bei Hemiprothese [4].

## Chirurgische Zugangswege

Gemäß internationalen Registerdaten werden in der Primärendoprothetik am häufigsten der posteriore, der laterale und zunehmend auch der direkte anteriore Zugang verwendet [16]. In der Revisionsendoprothetik, insbesondere bei PPAF, ist der primäre Zugangsweg nur dann wiederverwendbar, wenn eine adäquate Exposition der Fraktur möglich ist und sich dieser sicher erweitern lässt. Die Wahl des Zugangs bei PPAF richtet sich nach dem Frakturmuster, der notwendigen Exposition der betroffenen Strukturen und der Notwendigkeit der Revision der Prothesenkomponenten.

Da in den meisten Fällen der hintere Pfeiler betroffen ist, erlaubt ein primär anteriorer Zugang meist keine ausreichende Exposition. Ein zusätzlicher posteriorer Zugang mit Darstellung des Hinterpfeilers kann sinnvoll sein, selbst wenn die Primärprothese über einen anterioren oder lateralen Zugang implantiert wurde [4, 17]. Bei zusätzlichem Bedarf an direkter Inspektion der Pfannen- oder Femurkomponente kann alternativ eine Trochanter-Osteotomie durchgeführt werden, insbesondere bei kombinierten Frakturen oder komplexen Revisionssituationen [18, 19].

Liegen Frakturen des vorderen Pfeilers oder der quadrilateralen Fläche vor, werden vorrangig anteriore, intrapelvine, extraperitoneale Zugänge verwendet. Der ilioinguinale Zugang erlaubt die Darstellung des vorderen Pfeilers, wobei die Strukturen des Leistenkanals mobilisiert werden müssen; dies ist mit erhöhter Zugangsmorbidität und erhöhtem Aufwand verbunden [20]. Der modifizierte Stoppa-Zugang vermeidet die Dissektion dieser Strukturen, gilt als weniger invasiv und ermöglicht zusätzlich eine direkte Einsicht auf die quadrilaterale Fläche. Er ist besonders geeignet bei medialen Frakturen mit Beteiligung der quadrilateralen Fläche und Impression des Pfannendachs [21]. In bis zu 93 % der Fälle wird der Stoppa-Zugang mit einer zusätzlichen Inzision für das erste Fenster des ilioinguinalen Zugangs kombiniert, um die Beckenschaufel besser darzustellen und Schrauben in den hinteren Pfeiler einzubringen [22]. Der Pararectus-Zugang adressiert dieses Problem und erlaubt sowohl die Darstellung und Manipulation des vorderen Pfeilers und der quadrilateralen Fläche als auch die Platzierung von Schrauben weit posterior im Bereich des hinteren Pfeilers bzw. des posterioren Beckenrings ohne zusätzliche Inzision [23].

## Behandlungsalgorithmus

**Abb. 1:**
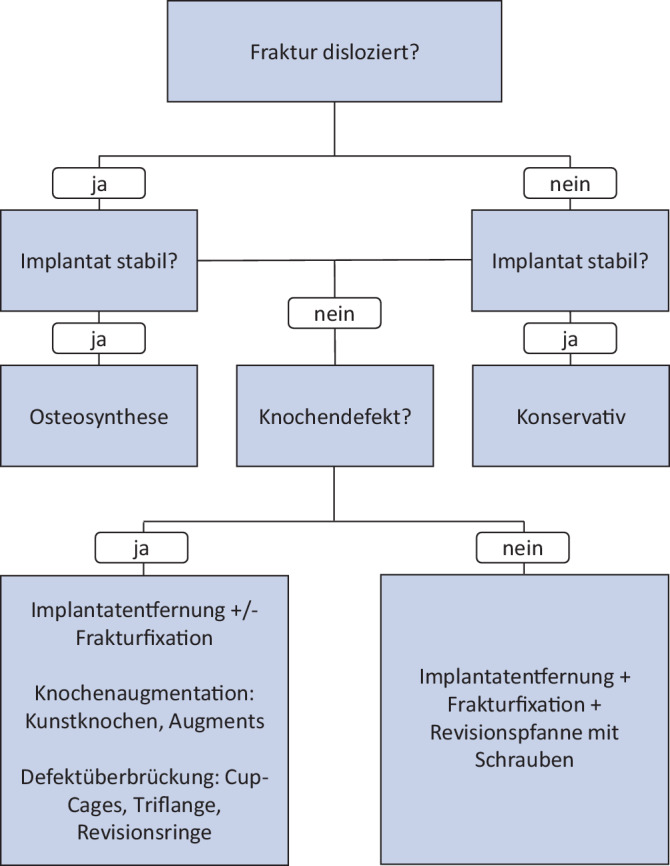
Behandlungsalgorithmus für periprothetische Acetabulumfrakturen. (Nach Acharya und Elnahal [7])

Für die Wahl der Behandlungsstrategie müssen die morphologischen Frakturmerkmale, einschließlich des Ausmaßes der Dislokation, sowie die Implantatstabilität und Knochendefekte berücksichtig werden [7]. Ein Vorschlag für einen Behandlungsalgorithmus ist in Abb. 1 ersichtlich. Das ausgewählte Verfahren muss in den Kontext von patientenbezogenen und weiteren frakturspezifischen Faktoren wie Alter, Begleiterkrankungen, Zeitpunkt der Fraktur nach der Implantation gesetzt werden.

### Undislozierte Fraktur, stabiles Implantat

**Abb. 2:**
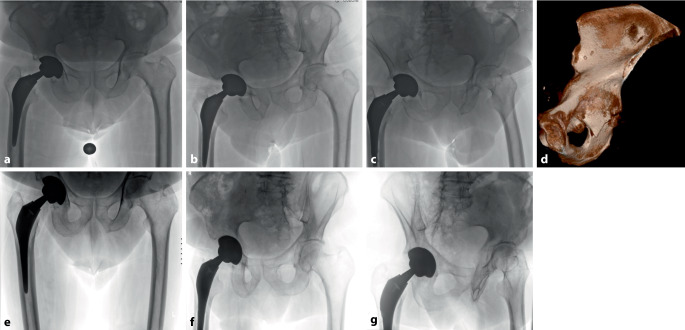
Aufnahmen einer 74-jährigen Patientin. **a**–**d** Rechtsseitige periprothetische Hinterpfeiler-Acetabulum-Fraktur und stabile Prothese nach Sturz aus Körperhöhe; **e**-**g** Verlaufskontrolle nach 6 Monaten konservativer Therapie mit vollständig konsolidierter Fraktur und stabilem Implantat (**a**, **e** a‑p.; **b**, **f** Ala, rechts; **c**, **g** Obturator, rechts; **d** CT-basierte 3D-Rekonstruktion mit Darstellung der Fraktur, Blick von intrapelvin)

Nichtdislozierte PPAF mit stabilem Implantat, entsprechend Typ IA oder IIIA nach Paprosky und Della Valle, können konservativ behandelt werden, mit Sohlenkontakt oder Entlastung für 6 bis 8 Wochen (Abb. 2; [6]).

### Dislozierte Fraktur, stabiles Implantat

**Abb. 3:**
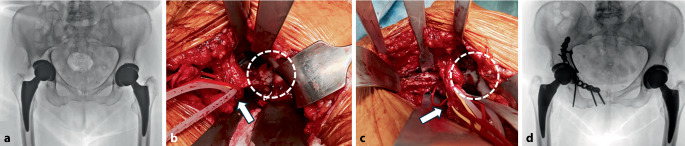
Aufnahmen einer 89-jährigen Patientin nach Sturz aus Körperhöhe. **a** Rechtsseitige periprothetische transverse Fraktur des Acetabulums mit stabilem Implantat. **b** Darstellung der Fraktur (*weißer Kreis*) über den Pararectus-Zugang. Distal davon verlaufen die Vasa iliaca externa (*weißer Pfeil*). **c** Reponierte Fraktur. **d** Verlaufskontrolle 2 Jahre postoperativ mit konsolidierter Fraktur und weiterhin stabilem Implantat nach Fixation mit einer suprapektinealen Platte, wobei der Hinterpfeiler durch Schrauben von anterior mitgefasst ist

Bei dislozierten Frakturen mit stabiler Pfannenkomponente und mehr als 50 % knöcherner Abstützungsfläche ist eine alleinige Frakturversorgung mithilfe der offenen Reposition und internen Fixation ohne Implantatwechsel möglich. Frakturen des hinteren Pfeilers nach primärer Implantation über einen posterioren Zugang können über denselben Zugang versorgt werden. Bei Frakturen des vorderen Pfeilers oder der quadrilateralen Fläche ist ein anteriorer Zugang (z. B. Pararectus-Zugang) erforderlich; dieser erlaubt die Beurteilung der Implantatstabilität von intrapelvin durch die Fraktur (Abb. 3; [5]).

### Dislozierte Fraktur, instabiles Implantat, kein Knochendefekt

Bei dislozierten PPAF, die intraoperativ im Rahmen der Implantation erkannt werden (z. B. Typ IB nach Paprosky und Della Valle), ist zunächst die Pfannenkomponente zu entfernen und die Fraktur vollständig darzustellen [6, 7]. Die Beurteilung erfolgt mithilfe der intraoperativen Bildgebung sowie durch Prüfung der Stabilität des anterioren und posterioren Pfeilers. In diesen Fällen sollte vor der Implantation der Pfanne eine offene Reposition und Fixation erfolgen.

Bei pelviner Diskontinuität ohne ausgeprägten Knochendefekt (Typ IIIa oder Va nach Paprosky und Della Valle) ist die Kompressionsosteosynthese des Hinterpfeilers mithilfe einer Rekonstruktionsplatte eine etablierte Therapieoption; insbesondere, wenn die Frakturen intraoperativ während der Pfannenimplantation erkannt werden [9]. Soll zusätzlich der vordere Pfeiler adressiert werden, ist auch eine perkutane Verschraubung bei lateralem Zugangsweg möglich. Eine zusätzliche Spongiosaplastik kann die knöcherne Konsolidierung fördern. Bei ausreichendem Knochenlager wird eine zementfreie, hemisphärische Revisionspfanne implantiert; diese wird über Schrauben fixiert. Sie übernimmt eine stabilisierende Funktion und ermöglicht eine sekundäre Osseointegration [6, 9]. Alternativ können verschraubte Revisionsringe mit zementiertem Inlay verwendet werden [24].

Postoperative Frakturen mit instabilem Implantat werden mit Pfannenwechsel mit/ohne Frakturfixation behandelt

**Abb. 4:**
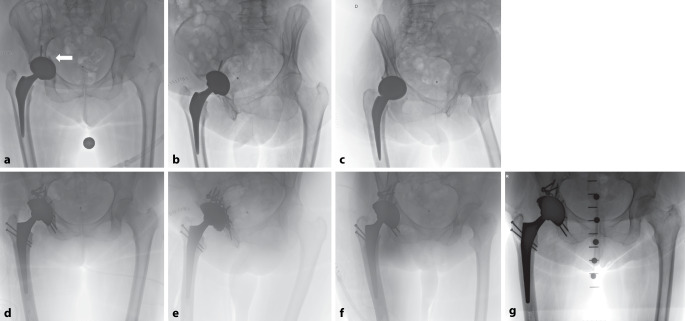
Aufnahmen einer 75-jährigen Patientin mit Sturz aus Körperhöhe 2 Wochen nach Implantation einer primären Hüfttotalendoprothese und Zuzug einer rechtsseitigen periprothetischen Acetabulumfraktur des Hinterpfeilers (**a** *weißer Pfeil*). Der Vorderpfeiler ist intakt; zudem findet sich intraoperativ ein lockerer Schaft: **a**–**c** Darstellung in a.-p.-, Ala- und Obturator-Aufnahmen. **d**–**f** Postoperative Bildgebung nach Revision mit Osteosynthese des Hinterpfeilers, Graft Impaction, Implantation einer verschraubten Revisionspfanne und Schaftwechsel (aufzementiert) über eine Trochanter-Osteotomie. **g** Verlaufskontrolle nach 2 Jahren

**Abb. 5:**
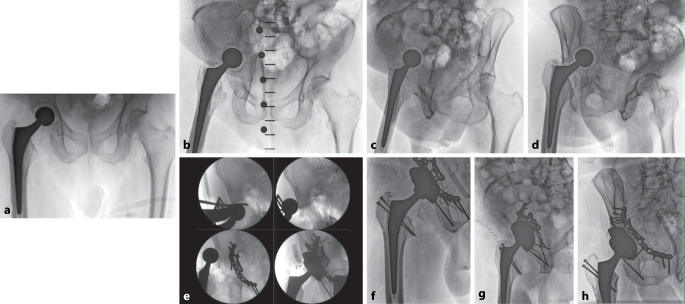
Aufnahmen eines 73-jährigen Patienten. **a** Initiales postoperatives Röntgen nach rechtsseitiger Implantation einer Hüfttotalendoprothese aufgrund einer medialen Schenkelhalsfraktur. Auffällig ist eine Kranialisation des Drehzentrums bei exzessivem Reaming. **b**–**d** Pfannenprotrusion mit pelviner Diskontinuität nach Sturz (oder Sturz bei Spontanfraktur) während des stationären Aufenthalts, Becken a.-p., Ala- und Obturator-Aufnahmen. **e** Intraoperative Bilder. Revision nach dem Prinzip der A‑Rahmen-Konstruktion über kombinierten Zugang: Pararectus-Zugang zur Osteosynthese des Vorderpfeilers mithilfe einer suprapektinealen Platte mit Eröffnung des vorbestehenden anterolateralen Zugangs zur Bergung der Pfanne, anschließend Zugang über Trochanter-Osteotomie zur Osteosynthese des Hinterpfeilers mithilfe einer Rekonstruktionsplatte bei Fraktur der Hinterwand mit kavitärem Defekt am kranialen Pfannenrand sowie Implantation einer Revisionspfanne mit Augment. **f**–**h** Postoperative Aufnahmen, Hüfte a.-p., Ala und Obturator

Bei postoperativen Frakturen mit instabilem Implantat (z. B. Typ IIIB nach Paprosky und Della Valle) ist meistens eine operative Versorgung erforderlich [25]. Das Frakturmuster nach der Letournel-Klassifikation dient als Grundlage für die Operationsplanung. Durch eine Osteosynthese kann der Pfanne ausreichende Stabilität verliehen und eine Deeskalation der Implantatauswahl ermöglicht werden [19]. Bei Beteiligung des Hinterpfeilers kann, wie oben erläutert, verfahren werden (Abb. 4). Ist der vordere Pfeiler beteiligt, kann eine suprapektineale Platte zur Frakturfixation verwendet werden. Sie stabilisiert gleichzeitig die quadrilaterale Fläche und verhindert eine mediale Migration der Pfanne [26]. Das Prinzip der Osteosynthese in diesen Fällen besteht in einer rahmenartigen Stabilisierung nach dem Konzept der sog. A‑Rahmen-Konstruktion (Abb. 5). Der vordere und hintere Pfeiler werden fixiert, und die verschraubte Pfannenkomponente übernimmt die verbindende Funktion zwischen beiden Strukturen [27].

### Dislozierte Fraktur, instabiles Implantat, ausgeprägter Knochendefekt

Das Cup-Cage-Konstrukt ist ein etabliertes Verfahren zur Versorgung pelviner Diskontinuitäten mit ausgeprägtem Knochendefekt (z. B. Typ Vb nach Paprosky und Della Valle, [9, 24]). Dabei wird eine unzementierte, poröse Revisionspfanne implantiert [19]. Häufig kommt eine „Jumbo-Pfanne“ zum Einsatz, definiert nach von Roth et al. als acetabuläre Komponente [6]. Die Pfanne wird press-fit im Becken verankert und mit einem abstützenden Revisionsring, der eine zusätzliche Schraubenverankerung in Os ilium und im Os ischii erlaubt, ergänzt. Der Ring dient zur Überbrückung knöcherner Defekte und stellt die primäre mechanische Stabilität sicher. Größere Defekte können vor der Pfannenimplantation mit einem Spongiosa-Graft oder modularen Augmenten ergänzt werden [19]. Cup-Cage-Konstrukte erzielen im Langzeitverlauf (bis zu 15 Jahre) Überlebensraten von 73–84 %, bei Komplikationsraten von 7–11 % für Luxationen, 9–13 % für Infektionen und 4–6 % für aseptische Lockerungen [28, 29].

Die Distraktionstechnik erzielt die initiale Stabilisierung und indirekte Reposition der Azetabulumfraktur

Alternativ kann die Distraktionstechnik angewendet werden [9]. Das Acetabulum wird sequenziell überreamt, bis die anterior-superioren und posterior-inferioren Randbereiche angefrischt sind. Anschließend wird eine hemisphärische Pfannenkomponente mit einem um 6–8 mm größeren Durchmesser als der letzte Reamer implantiert [6, 9]. Durch das Einbringen entsteht eine Distraktion im Bereich der Diskontinuität, die zu einer initialen Stabilisierung durch Press-fit sowie einer indirekten Reposition des Beckens infolge ligamentotaktischer Spannung führt [6]. Die Pfanne überbrückt den Defektbereich und verbindet die kranialen und kaudalen Anteile funktionell. Zusätzlich kann die Fixation mit multiplen Schrauben erfolgen [9]. Ein Follow-up von 2 bis 7 Jahren zeigte für die Distraktionstechnik niedrige Komplikationsraten, wobei aseptische Lockerungen mit Pfannenmigration die häufigsten Komplikationen darstellten (3–5 %, [6, 9, 30]).

Bei destruierendem Knochendefekt, der eine konventionelle Verankerungen nicht erlaubt, kann ein Implantat mit zusätzlichen Laschen verwendet oder auf einen individuell gefertigten Beckenteilersatz zurückgegriffen werden. Letzterer basiert auf CT-Planung und wird in der verbleibenden Knochensubstanz fixiert [9]. Die Überlebensraten dieser Implantate sind mit 81–90 % hoch, bei mittelfristigem Follow-up bis zu 10 Jahren. Die Komplikationsrate war erhöht, insbesondere die Dislokationsraten bis zu 25 % bei mehrfach voroperierten Patienten [9]. Limitierend kann die Verfügbarkeit in der akuten Situation sein.

## Fazit für die Praxis

- Periprothetische Acetabulumfrakturen (PPAF) sind selten, werden aber mit steigender Zahl der Hüfttotalendoprothesenimplantationen häufiger werden.
- Die Versorgung erfordert eine differenzierte Analyse von Frakturtyp, Implantatstabilität und Patientenfaktoren.
- Die Prinzipien der Frakturstabilisierung nach Letournel (anteriorer/posteriorer Pfeiler) sind konsequent anzuwenden.
- Eine stabile Fixation der Pfanne muss angestrebt werden. Eine zusätzliche osteosynthetische Stabilisierung kann die Primärstabilität verbessern und den Bedarf an komplexen Implantatsystemen reduzieren.
- Unfallchirurgische und orthopädische Expertise sind erforderlich.
